# High expression of tumour-associated trypsin inhibitor correlates with liver metastasis and poor prognosis in colorectal cancer

**DOI:** 10.1038/sj.bjc.6605047

**Published:** 2009-04-21

**Authors:** A Gaber, M Johansson, U-H Stenman, K Hotakainen, F Pontén, B Glimelius, A Bjartell, K Jirström, H Birgisson

**Affiliations:** 1 Department of Laboratory Medicine, Center for Molecular Pathology, Malmö University Hospital, Lund University, Malmö, Sweden; 2 Department of Clinical Chemistry, University of Helsinki, Helsinki, Finland; 3 Department of Genetics and Pathology, Rudbeck Laboratory, Uppsala University, Uppsala, Sweden; 4 Department of Oncology, Radiology and Clinical Immunology, University of Uppsala, Uppsala, Sweden; 5 Department of Oncology and Pathology, Karolinska Institute, Stockholm, Sweden; 6 Division of Urological Cancers, Department of Clinical Sciences, University Hospital Malmö, Lund University, Malmö, Sweden; 7 CREATE Health, Lund University, Malmö, Sweden; 8 Department of Surgery, University of Uppsala, Uppsala, Sweden; 9 Centre for Clinical Research, Uppsala University, Central Hospital, Västerås, Sweden

**Keywords:** colorectal cancer, TATI, prognosis, liver metastasis

## Abstract

Increased expression of tumour-associated trypsin inhibitor (TATI) in tumour tissue and/or serum has been associated with poor survival in various cancer forms. Moreover, a proinvasive function of TATI has been shown in colon cancer cell lines. In this study, we have examined the prognostic significance of tumour-specific TATI expression in colorectal cancer, assessed by immunohistochemistry (IHC) on tissue microarrays (TMAs) with tumour specimens from two independent patient cohorts. Kaplan–Meier analysis and Cox proportional hazards modelling were used to estimate time to recurrence, disease-free survival and overall survival. In both cohorts, a high (>50% of tumour cells) TATI expression was an independent predictor of a significantly shorter overall survival. In cohort II, in multivariate analysis including age, gender, disease stage, differentiation grade, vascular invasion and carcinoembryonal antigen (CEA), high TATI expression was associated with a significantly decreased overall survival (HR=1.82; 95% CI=1.19–2.79) and disease-free survival (HR=1.56; 95% CI=1.05–2.32) in curatively treated patients. Moreover, there was an increased risk for liver metastasis in both cohorts that remained significant in multivariate analysis in cohort II (HR=2.85; 95% CI=1.43–5.66). In conclusion, high TATI expression is associated with liver metastasis and is an independent predictor of poor prognosis in patients with colorectal cancer.

Tumour invasion of surrounding tissue requires degradation of the basal membrane and extracellular matrix (Kohn and Liotta, 1995). Matrix metalloproteinases (MMPs) and matrix serine proteases (MSPs) are two groups of proteolytic enzymes that are proposed to be the most important for tumour progression (Stenman, 1990; Mignatti and Rifkin, 1993; Nelson *et al*, 2000). Trypsin is a potent MSP protease that hydrolyses a variety of proteins and activates other MSPs and MMPs (Koivunen *et al*, 1991; Yamamoto *et al*, 2003). Four identical isoforms of trypsinogen have been described in human tissue and these isoforms are homologous (Emi *et al*, 1986; Tani *et al*, 1990; Wiegand *et al*, 1993; Koshikawa *et al*, 1998). In the gastrointestinal tract, the main function of the trypsin is to break down dietary proteins. It is expressed by many tumours and also plays a significant role in tumour invasion (Koivunen *et al*, 1991; Moilanen *et al*, 2003; Paju *et al*, 2004). Immunohistochemical (IHC) expression of trypsin in colorectal cancer (CRC) correlates with unfavourable clinicopathological characteristics and shortened survival (Yamamoto *et al*, 2003).

The tumour-associated trypsin inhibitor (TATI), synonymous with serine protease inhibitor Kazal type 1 (SPINK1) and pancreatic secretory trypsin inhibitor (PSTI; Huhtala *et al*, 1982), balances and inhibits trypsin specifically and may thus reduce tissue destruction (Wiksten *et al*, 2005). It is also a weak inhibitor of other serine proteinases (Fritz *et al*, 1967; Huhtala *et al*, 1982; Turpeinen *et al*, 1988). *In vitro*, TATI increases cell migration and plays a role in tissue repair (Stenman, 1990). It has been suggested that expression of TATI and trypsin is balanced in normal tissue, but this balance could be disrupted during tumour progression (Hotakainen *et al*, 2006).

Tumour-associated trypsin inhibitor expression has been associated with impaired survival in several forms of cancer (Stenman, 2002; Paju *et al*, 2004; Lee *et al*, 2007) but not in gastric cancer, where it is believed to have a natural function of protecting the mucosa from proteolytic degradation (Freeman *et al*, 1990; Playford *et al*, 1991; Marchbank *et al*, 1996, 1998; Wiksten *et al*, 2005). However, in most cancer forms, TATI and trypsin are coexpressed and show similar and adverse associations to disease outcome (Paju *et al*, 2004; Hotakainen *et al*, 2006).

Elevated serum TATI has been shown to be a prognostic marker for ovarian cancer (Venesmaa *et al*, 1994, 1998), kidney cancer (Paju *et al*, 2001) and bladder cancer (Kelloniemi *et al*, 2003). In ovarian carcinoma, TATI expression, both in tissue and serum, has been associated with a shorter survival and high tissue expression was the most useful prognostic factor (Huhtala *et al*, 1982; Paju *et al*, 2004).

Coexpression of trypsin and TATI has previously been found at both mRNA and protein level in CRC (Solakidi *et al*, 2003), but we are not aware of any published reports on the association between expression of TATI in tumour tissue and survival in patients with CRC. However, a recent study showed that TATI is associated with autocrine induction and metastasis in colon cancer cells (Gouyer *et al*, 2008).

The purpose of this study was to analyse the association between IHC expression of TATI in CRC and clinicopathological parameters, its recurrence and survival.

## Patients and methods

### Cohort I

Cohort I includes 118 cases of CRC, 61 (52%) women and 57 (48%) men, diagnosed at the Department of Pathology, Malmö University Hospital, between January 1999 and March 2002. This cohort was designed as an initial screening cohort for tissue biomarkers of potential diagnostic or prognostic relevance. Therefore, the cases were selected to obtain an equal distribution of disease stages I–III, with 35 patients in stage I, 42 patients in stage II, 35 patients in stage III and 6 patients in stage IV. Median age at diagnosis was 75 years (range: 32–88; mean 73), and after a median follow-up of 85 months (0–115) (mean 56), 54 patients (46%) were alive and 64 (54%) were dead. Information on treatment was not available for this cohort. Data on overall survival (OS) were collected from the population register. Approval was obtained from the Ethics committee at the Lund University (cohort I, ref no. 447–07), whereby informed consent was deemed not to be required other than by the opt-out method.

### Cohort II

The second cohort, used as a validation cohort, consists of 320 prospectively collected patients undergoing elective surgery for CRC at the Central District Hospital in Västerås, Sweden, between June 2000 and December 2003, 277 (87%) of which were treated surgically with curative intent. Information on cancer recurrence, death and cause of death were obtained by matching with the Regional Oncology Registry and from the hospital records. Median follow-up time was 6 years (4–7) for surviving patients. Recurrent disease was reported for 54 (19%) of curatively treated patients, and 119 (37%) patients died during the study period. Preoperative radiotherapy was given to 84/108 patients with rectal cancer. All patients <75 years with colon cancer stage III (*n*=36) and 22 of 29 rectal cancer patients as well as some patients with high risk (T4, low differentiation) stage II disease (13/71) received adjuvant chemotherapy. Palliative chemotherapy was given in 23 of 27 patients <75 years with stage IV disease. More than 12 lymph nodes were examined in 224 (70%) of 320 patients and in 96 (73%) of 132 patients with stage II disease. Ethical approval was obtained from the Ethics committee at Uppsala University (cohort II, ref no. 00–001). All patients included in this cohort gave their informed consent for participation in the study.

### Tissue microarray (TMA) construction

Tissue microarrays were constructed as described earlier (Kononen *et al*, 1998). In brief, 2 × 1 mm cores from areas representative of invasive cancer were sampled for each case and mounted in a recipient block using a manual arraying device (MTA-1; Beecher Instruments, Hartland, WI, USA). In addition, a number of samples from normal colonic mucosa, adenomas and lymph node metastases were included in the TMA from cohort II.

### Immunohistochemistry

In all, 4 *μ*m sections were dried, deparaffinised, rehydrated, and heat-mediated antigen retrieval was carried out by boiling under pressure in DAKO Target Retrieval Buffer (DAKO, Glostrup, Denmark; pH 9.0). Immunohistochemistry was carried out in the DAKO Techmate 500 system (DAKO) using a monoclonal anti-TATI antibody (6E8) diluted 1 : 500 as described earlier (Osman *et al*, 1993).

Tumour-associated trypsin inhibitor was expressed in the cytoplasm and the percentage of positive tumour cells in each core was estimated and assigned values of 0, 5 or multiples of 10%. The intensity of the expression was assigned a value of 0, 1, 2 or 3. In cases where both tissue cores were present, both the score from the core with the highest percentage of positive cells/‘best score’ (Tomlins *et al*, 2008) and mean score were denoted. Samples from adjacent normal mucosa and adenomas were scored in the same fashion. The IHC staining was evaluated independently by two of the authors (AG and KJ) who were blinded to clinical and outcome data. Scoring differences were discussed to reach consensus.

### Statistics

The *χ*^2^ and Spearman's correlation tests were used for comparison of TATI expression and relevant patient and tumour characteristics. The Kaplan–Meier method and log-rank test were used to estimate disease-free survival (DFS), time to recurrence (TTR) and OS. End points were defined as recommended by Punt *et al* (2007); OS was measured from the date of surgery to the date of death; the observations were censored at the end of the study period (1 November 2008). Disease-free survival was measured in curatively treated patients from the date of surgery to the date of second cancer, recurrence or death from any cause. Time to recurrence was measured in curatively treated patients from the date of surgery to the date of locoregional recurrence, distant metastases or to the date of death in CRC; the observations were censored at the end of the study period or at the date of death in non-CRC. A Cox proportional hazards model/hazard ratios (HRs) was used for estimation of relative risks in both univariate and multivariate analyses, and adjusted for age, gender, disease stage, differentiation grade and lymphatic or vascular invasion. All statistical tests were two-sided and *P*-values <0.05 were considered significant. Calculations were carried out with either SPSS15.0 (SPSS Inc., Chicago, IL, USA) or Statistica 7 (Stat Soft Inc., Tulsa, OK, USA).

## Results

### Tumour-associated trypsin inhibitor expression in normal colonic mucosa, cancer and metastases

**Figure 1:**
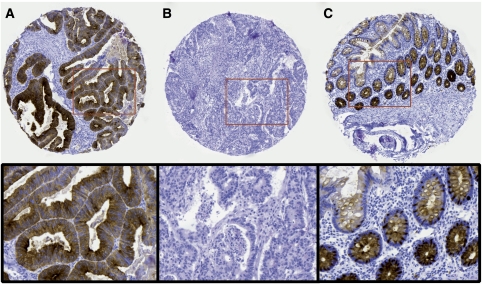
Immunohistochemical images of invasive cancer with high (**A**) and low (**B**) tumour-associated trypsin inhibitor (TATI) score and expression of TATI in adjacent non-malignant mucosa (**C**).

Tumour-associated trypsin inhibitor was expressed in the cytoplasm both in the normal mucosa, the primary cancer and metastases (Figure 1). The overall staining distribution (best score) in cancer cells was similar in cohorts I and II (data not shown). There was no obvious heterogeneity in TATI expression between the paired tissue cores from each individual tumour, that is, no difference between mean and best scores (data not shown). Using cohort I as a test cohort to construct a dichotomised variable for defining TATI high and low cases, we compared different thresholds (1, 25, 50 and 75% positive cells) with respect to their impact on survival. High TATI expression was associated with a shorter survival for all cutoffs tested (data not shown), but the most significant separation was observed at 50%. The staining intensity had no impact on survival. By this approach, the proportion of cases with low and high expression was similar in both cohorts with 24 cases (20.3%) in cohort I and 66 cases (19.9%) in cohort II exhibiting high TATI expression. No differences were seen in the expression of TATI best score in different preoperative radiation therapy groups in rectal cancer patients (data not shown).

### Correlation between TATI expression and clinicopathological parameters

**Figure 2:**
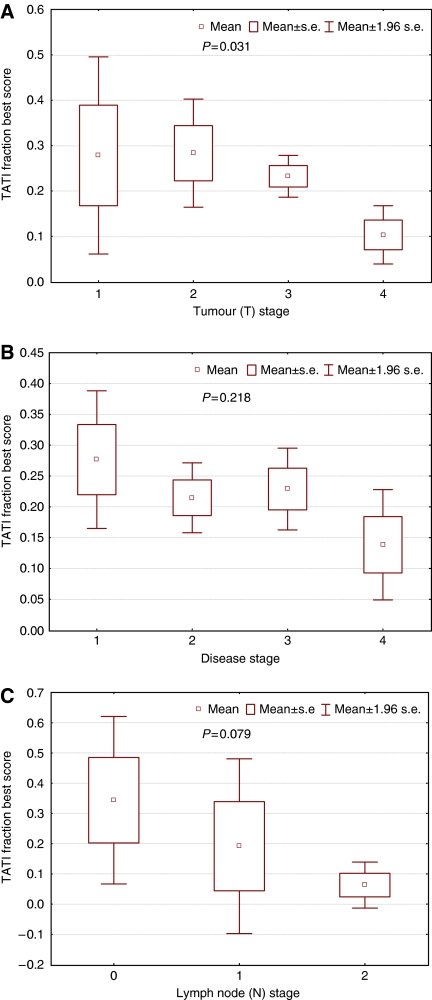
Tumour-associated trypsin inhibitor (TATI) best score for fraction of immunoreactivity in primary tumour tissue from patients with colorectal cancer and its relation to tumour (T) (**A**), disease (**B**) and to lymph node (N) stages (**C**). Boxes indicate mean (small central box) and mean ±1 s.e. (larger box). Whiskers indicate mean ±1.96 times s.e. Kruskal–Wallis test was used for comparison.

The distribution of tumours with low and high TATI expression in relation to tumour and patient characteristics is shown in Table 1. In cohort I, high TATI expression was only significantly associated with age (*P*=0.008). In cohort II, lower expression was seen with advanced tumour (T), lymph node (N) and disease stage, also visualised in Figure 2. There was a significant association between a lower TATI fraction (0–50%) and higher tumour stage (*P*=018). In cohort II, TATI was expressed in 89 (86%) of 104 samples from benign mucosa and in 12 (18%) of 67 lymph node metastases. When right and left colonic tumours were compared with left colonic cancers, defined as tumours in the splenic flexure to the rectum, a trend for lower expression of TATI best score was seen in left colonic cancers. Tumour-associated trypsin inhibitor best score ⩽50 was seen in 141 of 188 (0.75%) left colonic cancers compared with 100 out of 121 (0.83%) right colonic cancers (*P*=0.113, *χ*^2^-test).

### Tumour-associated trypsin inhibitor expression in relation to survival and metastasis

In cohort I, tumours with more than 50% TATI-expressing tumour cells had a significantly shorter OS (HR=2.42; 95% CI= 1.38–4.26, *P*=0.002). This association remained significant in multivariate analysis after adjustment for established clinicopathological parameters (HR=1.80; 95% CI= 0.99–3.27; *P*=0.05). There was a significant association with the appearance of liver metastases (HR=3.97; 95% CI=0.99–15.88; *P*=0.05) in univariate, but not in multivariate analysis, including age, gender, disease stage, differentiation grade and vascular invasion (HR= 3.69; 95% CI= 0.87–15.67; *P*=0.08).

**Figure 3:**
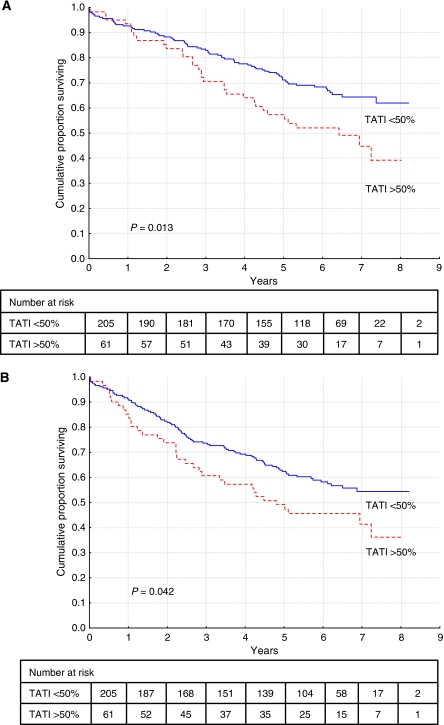
Overall survival (**A**) and disease-free survival (**B**) in curatively treated patients with colorectal cancer in cohort II according to high (*n*=61) *versus* low (*n*=205) TATI best score (cutoff =50% positive cells).

In cohort II, patients with high TATI score had a significantly shorter OS and DFS, compared to those with low fraction, in curatively treated patients (Figure 3). This association remained significant in multivariate analysis, including adjustment for age, gender, disease stage, differentiation grade, vascular invasion and carcinoembryonal antigen (CEA; Table 2).

**Figure 4:**
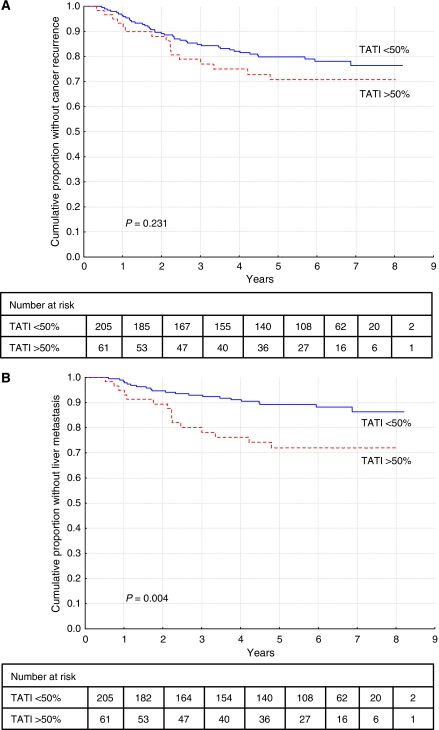
Time to recurrence (**A**) and recurrence of liver metastasis (**B**) in curatively treated patients with colorectal cancer in cohort II according to high (*n*=61) *versus* low (*n*=205) TATI best score (cutoff=50% positive cells).

Furthermore, patients with a high fraction of TATI-positive cells in primary tumour tissue tended to have increased risk of cancer recurrence (Figure 4a) and an increased risk of liver metastasis (Figure 4b), and this was significant also in multivariate analysis (HR=2.85; 95% CI=1.43–5.66).

As mentioned earlier most of the patients (*n*=40) with metastatic disease at diagnosis had TATI best score <50% (*n*=34; 85%) (Figure 2b); however, there was a trend towards more synchronous liver metastases in those with TATI >50% (5 of 6; 83%) compared with TATI<50% in primary tumour tissue (16 of 34; 47%; *P*=0.101).

## Discussion

Our results show that tumour-specific TATI expression is associated with shorter survival of patients with CRC. These findings were observed in two independent patient cohorts. These results are in line with recent *in vitro* data (Gouyer *et al*, 2008). Furthermore, in both cohorts, there was a significant association between TATI expression and metastasis to the liver and in cohort II; representing a larger number of patients, this association remained significant in multivariate analysis.

An elevated TATI expression has, with few exceptions, been associated with a more aggressive tumour phenotype and poor clinical outcome in several forms of cancer (Paju *et al*, 2004, 2007; Hotakainen *et al*, 2006; Lee *et al*, 2007; Tomlins *et al*, 2008). Given the frequently observed coexpression of TATI and trypsin in cancer, it can be hypothesised that increased levels of TATI reflect a simultaneous elevation of trypsin and, hence, increased propensity of the tumour cells to invade and spread. However, in prostate cancer, TATI may actually be an independent mediator of aggressive disease (Tomlins *et al*, 2008). The mechanisms by which TATI is associated with aggressive cancer need to be further explored. Although protease inhibitors could be expected to control the invasion-promoting effects of various proteases, there is increasing evidence showing that overexpression of various protease inhibitors results in enhanced malignancy of cancer cells. For instance, a number of studies have shown that increased serum concentrations of tissue inhibitor of metalloproteinases 1 (TIMP-1), a major inhibitor of metalloproteinases, often is associated with a poor clinical outcome in various cancer forms (Ree *et al*, 1997; Holten-Andersen *et al*, 1999; Schrohl *et al*, 2004; Yukawa *et al*, 2004). Similarly, another protease inhibitor, the plasminogen activator inhibitor 1 (PAI-1) has been shown to be associated with poor prognosis in breast cancer (Grondahl-Hansen *et al*, 1993), lung cancer (Pedersen *et al*, 1994) and CRC (Nielsen *et al*, 1998). Overexpression of secretory leukocyte protease inhibitor (SLPI), which also inhibits trypsin, increases the malignant properties of lung cancer cell lines (Devoogdt *et al*, 2003).

So far, the mechanisms by which TATI is associated with tumour aggressiveness are unknown, but because no TATI receptor has been identified and TATI is a very specific trypsin inhibitor (Turpeinen *et al*, 1988), it is tempting to speculate that TATI exerts its effect through modulation of trypsin activity. Trypsin may affect various cellular functions, for example, proliferation and invasion, by activating protease-activated receptors (PARs) and especially PAR-2 (Soreide, 2008).

The complexity of the interplay and diverging actions of proteases and their inhibitors in human cancer is further illustrated by the fact that experimental targeted therapies with synthetic proteinase inhibitors have not proven effective and have, in some trials, even elicited poorer results and more adverse effects than standard chemotherapy (Kruger *et al*, 2001; Coussens *et al*, 2002; Moore *et al*, 2003).

Furthermore, treatment with the synthetic MMP inhibitor, batimastat, has been shown to make breast cancer and lymphoma cells more prone to form liver metastases in mouse models (Kruger *et al*, 2001). In addition, batimastat treatment induced the expression of MMPs, metastasis-promoting and angiogenesis factors in the liver (Kruger *et al*, 2001). This is particularly interesting in the light of our findings of a significant association between TATI expression and metastasis to the liver. A link between TATI and metastatic disease has also been shown in CRC cell lines and downregulation of TATI resulted in a concomitant downregulation of several metastasis-associated genes (Gouyer *et al*, 2008).

Another explanation for the more malignant phenotype associated with TATI overexpression could be that an increased expression of proteinase inhibitors may help the tumour cells evade the immune response and make them better equipped for survival and distant spread. It has been suggested that the increased expression of proteinases associated with malignant disease may, to a large extent, be because of an immunological host response induced by the tumour (Nelson *et al*, 2000). For instance, in breast cancer cells, stromelysin-3, a proteinase from the MMPs family, has been found exclusively in stromal cells surrounding neoplastic invasive cells, but not non-invasive cells (Basset *et al*, 1990).

Tumour-associated trypsin inhibitor is believed to play a protective role in both gastric mucosa (Wiksten *et al*, 2005) and colonic mucosa (Soreide *et al*, 2006) and in accordance with these findings, we found that TATI was highly expressed in non-malignant colonic mucosa. It should, however, be pointed out that the non-malignant mucosa studied here had been sampled from areas adjacent to the tumour and, hence, it cannot be ascertained whether this truly reflects TATI levels in normal mucosa from healthy individuals.

In cohort II, there was a significant association between lower TATI expression and a more advanced tumour stage. Such an association has previously been found in a small cohort of bladder cancer (Hotakainen *et al*, 2006) where high TATI expression tended to be associated with a better prognosis. There was also a trend, however non-significant, towards a lower TATI expression in more advanced disease stages; but the proportion of synchronous liver metastases was higher in cases with TATI >50%.

Further studies are warranted to investigate which cutoffs are the most appropriate for defining high *versus* low TATI expression. In this study, we first analysed TATI expression in a retrospectively collected cohort and established a dichotomised variable with the best prognostic separation. This cutoff also proved to have a prognostic impact in a second cohort with prospectively collected tumour samples, in which the risk of sampling bias should be reduced. In neither of the cohorts did the staining intensity have any impact on prognosis, only the fraction of positive cells. As the staining intensity is often more easily affected by the variability of sample preparation, particularly the time of fixation in formalin, the fraction of positive cells might be a more reliable parameter with better reproducibility. Both parameters should, however, be taken into account in future studies evaluating TATI expression by IHC, and automated image analysis may be a more objective approach (Brennan *et al*, 2008; Rexhepaj *et al*, 2008). Other cutoffs, with or without taking the staining intensity into account, have been used in earlier studies on other cancer forms, (Paju *et al*, 2004; Tomlins *et al*, 2008).

It will also be of interest to investigate the utility of serum TATI as a prognostic marker and predictor of liver metastasis in CRC. However, TATI as well as CEA in serum has not proven to correlate with the absence or presence of metastases or monitoring of disease after surgery (Catarino and Conde, 1991; Pasanen *et al*, 1995).

In conclusion, our results show that tumour-specific overexpression of TATI contributes to a poor prognosis in CRC and seems to promote a tumour phenotype with predilection of liver metastasis *in vivo*. Thus, therapeutic targeting of TATI could prove to be an efficient strategy in the management of high-risk CRC patients.

## Figures and Tables

**Table 1:** Correlation betweeen TATI expression and clinicopathological parameters in two colorectal cancer cohorts

	**Cohort I**			**Cohort II**		
**TATI fraction**	**0–50%**	**>50%**		**0–50%**	**>50%**	
***n* (%)**	**90 (76.3)**	**24 (20.3)**	P-value*	**241 (78.0)**	**68 (22.0)**	P-value*
*Age*						
⩽75	50 (55.6)	6 (25.0)		143 (59.3)	33 (48.5)	
>75	40 (44.4)	18 (75.0)	0.008	98 (40.7)	35 (51.5)	0.112
						
*Gender*						
Female	49 (54.4)	9 (37.5)		120 (49.8)	32 (47.1)	
Male	41 (45.6)	158 (62.5)	0.143	121 (50.2)	36 (52.9)	0.691
						
*Tumour (T) stage*						
1				9 (3.7)	3 (4.4)	
2				28 (11.6)	12 (17.6)	
3				156 (64.7)	47 (69.1)	
4				48 (19.9)	6 (8.8)	0.142
						
*Lymph node (N) stage*						
0	50(55.6)	14(58.3)		138 (57.3)	42 (61.8)	
1	19(21.1)	6(25.0)		46 (19.1)	16 (23.5)	
2	8(8.9)	1(4.2)	0.65	57 (23.7)	10 (14.7)	0.302
No information	13(14.4)	3(12.5)				
						
*Disease stage*						
I	24 (26.7)	11 (45.8)		31 (12.9)	13 (19.1)	
II	35 (38.9)	5 (20.8)		101 (41.9)	26 (38.2)	
III	28 (31.1)	5 (20.8)		75 (31.1)	23 (33.8)	
IV	3(3.3)	3(12.5)	0.277	*34* (*14.1)*	*6* (*8.8)*	0.305
						
*Differentiation grade*						
High–moderate	73 (81.1)	21 (87.5)		186 (77.2)	55 (80.9)	
Poor	17 (18.9)	3 (12.5)	0.46	55 (22.8)	13 (19.1)	0.515
						
*Vascular invasion*						
No invasion	78 (86.7)	21 (87.5)		209 (86.7)	59 (86.8)	
Invasion	12 (13.3)	3 (12.5)	0.915	32 (13.3)	9 (13.2)	0.993
						
*CEA*						
<6 ng ml^−1^				158 (65.6)	53 (77.9)	
⩾6 ng ml^−1^				*73* (*30.3)*	*13* (*19.1)*	
No information				10 (4.1)	2 (2.9)	0.061

TATI=tumour-associated trypsin inhibitor.

* *χ*^2^-test of association was used for 2 × 2 tables and *χ*^2^-test for linear trend for tables with more than two rows and/or columns.

The number of cases with missing data is given for some variables, but these are not included in the analyses.

**Table 2:** The relative risks for death in curatively treated patients with colorectal cancer from cohort II

	**Relative risk for death (overall survival)**			**Relative risk for second cancer, recurrence or death to any cause in curatively treated patients (disease-free survival)**		
		**Univariate**	**Multivarite**		**Univariate**	**Multivariate**
	***n***	**HR (95% CI)**	**HR (95% CI)**	***n***	**HR (95% CI)**	**HR (95% CI)**
*Age at operation*						
Age <75 years	154	1.0 (ref)	1.0 (ref)	154	1.0 (ref)	1.0 (ref)
Age ⩾75 years	123	2.39 (1.62–3.52)	2.74 (1.83–4.09)	123	1.87 (1.32–2.65)	2.09 (1.46–3.0)
*Gender*						
Female	137	1.0 (ref)	1.0 (ref)	137	1.0 (ref)	1.0 (ref)
Male	140	0.78 (0.53–1.14)	0.74 (0.50–1.09)	140	0.81 (0.57–1.15)	0.79 (0.55–1.12 )
*Disease stage*						
Stage I	45	1.0 (ref)	1.0 (ref)	45	1.0 (ref)	1.0 (ref)
Stage II	131	1.09 (0.58–2.02)	1.18 (0.62–2.24)	131	1.57 (0.85–2.87)	1.64 (0.88–3.05)
Stage III	100	1.45 (1.07–1.97)	1.55 (1.13–2.13)	100	1.62 (1.20–2.19)	1.69 (1.24–2.31)
						
*Differentiation grade*						
High–moderate	221	1.0 (ref)	1.0 (ref)	221	1.0 (ref)	1.0 (ref)
Poor	56	1.40 (0.90–2.20)	1.36 (0.87–2.15)	56	1.38 (0.88–2.17)	1.30 (0.86–1.96)
						
*Lymphatic or vascular vessel invasion*						
No invasion	252	1.0 (ref)	1.0 (ref)	252	1.0 (ref)	1.0 (ref)
Invasion	25	1.68 (0.94–3.01)	1.64 (0.90–2.99)	25	1.93 (1.15–3.21)	1.73 (1.02–2.94)
						
*CEA*						
<6 ng ml^−1^	204	1.0 (ref)	1.0 (ref)	277	1.0 (ref)	1.0 (ref)
>6 ng ml^−1^	62	1.41 (0.92–2.17)	1.35 (0.88–2.09)	0	1.56 (1.06–2.29)	1.48 (1.00–2.20)
						
*TATI fraction of immunorectivity best score*						
<50%	205	1.0 (ref)	1.0 (ref)	205	1.0 (ref)	1.0 (ref)
>50%	61	1.73 (1.14–2.64)	1.82 (1.19–2.79)	61	1.52 (1.03–2.25)	1.56 (1.05–2.32)

CI=confidence interval; CEA=carcinoembryonal antigen; HR=hazard ratio; TATI=tumour-associated trypsin inhibitor.

Uni- and multivariate analyses, including age, gender, disease stage, differentiation grade, lymphatic or vascular vessel invasion, CEA and TATI fraction of immunoreactivity best score.
